# Expanding Our Horizons: The Process of Seeking Accreditation from AGEC

**DOI:** 10.1093/geroni/igaf122.2147

**Published:** 2025-12-31

**Authors:** Katarina Friberg-Felsted, Jacqueline Eaton, Kara Dassel, Sara Hart, Rebekah Perkins, Gail Towsley, Marla De Jong

**Affiliations:** University of Utah, Salt Lake City, Utah, United States; University of Utah, Salt Lake City, Utah, United States; University of Utah, Salt Lake City, Utah, United States; University of Utah, Salt Lake City, Utah, United States; University of Utah, Salt Lake City, Utah, United States; University of Utah, Salt Lake City, Utah, United States; University of Utah, Salt Lake City, Utah, United States

## Abstract

As the final part of the age-inclusivity initiative to enhance excellence in education at the University of Utah College of Nursing (CON), the Gerontology Interdisciplinary Program (GIP) sought to elevate the Master of Science in Gerontology (MSG) curriculum to a standard level of national accreditation. The GIP is unique in that it has a 43-year history as the only interdisciplinary degree housed in the College of Nursing (CON). Although the CON has a long tradition of offering accredited programs of nursing, we facilitated purposeful collaboration across programs to align standards and apply for inaugural accreditation of the MSG. The abstract presenter will transparently describe the process of working with the Accreditation for Gerontology Education Council (AGEC) to demonstrate how the MSG meets accreditation standards for gerontology education and how graduate students achieve gerontology competencies. Across two years, we conducted a program self-study, delineating our programmatic structure, student learning goals and outcomes, and ensuring alignment with Academy for Gerontology in Higher Education (AGHE) competencies. We established support documents such as evidence of competency alignment, program policies, and faculty credentials. Following submission of the self-study, we responded to review team feedback and prepared for a 2-day site visit. Multiple faculty dedicated substantial time to prepare materials; coordinated meetings with university leaders, community partners, and faculty; and hosted site visitors. Participating in the process of accreditation increased our collaboration across the College of Nursing, improved our visibility with university leadership, and augmented investment from community partners both internal and external to the university.

